# Tamoxifen for the management of breast events induced by non-steroidal antiandrogens in patients with prostate cancer: a systematic review

**DOI:** 10.1186/1741-7015-10-96

**Published:** 2012-08-28

**Authors:** Frank Kunath, Bastian Keck, Gerd Antes, Bernd Wullich, Joerg J Meerpohl

**Affiliations:** 1 German Cochrane Center, Institute of Medical Biometry and Medical Informatics, University Medical Center Freiburg, Berliner Allee 29, 79110 Freiburg/Br., Germany; 2 Department of Urology, University Clinic Erlangen, Krankenhausstr. 12, 91054 Erlangen, Germany; 3 Pediatric Hematology and Oncology, Center for Pediatrics and Adolescent Medicine, University Medical

**Keywords:** Prostatic neoplasms, Androgen suppression therapy, Gynecomastia, Tamoxifen, Systematic review, Meta-analysis

## Abstract

**Background:**

Tamoxifen has emerged as a potential management option for gynecomastia and breast pain due to non-steroidal antiandrogens, and it is considered an alternative to surgery or radiotherapy. The objective of this systematic review was to assess the benefits and harms of tamoxifen, in comparison to other treatment options, for either the prophylaxis or treatment of breast events induced by non-steroidal antiandrogens in prostate cancer patients.

**Methods:**

We searched CENTRAL, MEDLINE, EMBASE, reference lists, the abstracts of three major conferences and three trial registers to identify ongoing randomized controlled trials (RCTs). Two authors independently screened the articles identified, assessed the trial quality and extracted data. The protocol was prospectively registered (CRD42011001320; http://www.crd.york.ac.uk/PROSPERO).

**Results:**

Four studies were identified. Tamoxifen significantly reduced the risk of suffering from gynecomastia (risk ratio 9RR0 0.10, 95% CI 0.05 to 0.22) or breast pain (RR 0.06, 95% CI 0.02 to 0.17) at six months compared to untreated controls. Tamoxifen also showed a significant benefit for the prevention of gynecomastia (RR 0.22, 95% CI 0.08 to 0.58) and breast pain (RR 0.25, 95% CI 0.10 to 0.64) when compared to anastrozole after a median of 12 months. One study showed a significant benefit of tamoxifen for the prevention of gynecomastia (RR 0.24, 95% CI 0.09 to 0.65) and breast pain (RR 0.20, 95% CI 0.06 to 0.65) when compared with radiotherapy at six months. Radiotherapy increased the risk of suffering from nipple erythema and skin irritation, but there were no significant differences for any other adverse events (all *P *> 0.05).

**Conclusions:**

The currently available evidence suggests good efficacy of tamoxifen for the prevention and treatment of breast events induced by non-steroidal antiandrogens. The impact of tamoxifen therapy on long-term adverse events, disease progression and survival remains unclear. Further large, well-designed RCTs, including long-term follow-ups, are warranted. Also, the optimal dose needs to be clarified.

## Background

Prostate cancer growth is influenced by androgenic activity [1], and androgen suppression therapy is a non-curative therapeutic option for locally advanced, non-metastatic, lymph-node positive, symptomatic or asymptomatic metastatic prostate cancer either to slow progression or to palliate symptoms of the disease [2]. Current guidelines suggest surgical castration by bilateral orchiectomy and monotherapy with luteinizing hormone-releasing hormone (LHRH) agonists as the standard treatment for patients with advanced prostate cancer. Antiandrogens are recommended for short-term administration in patients receiving LHRH agonists and non-steroidal antiandrogen monotherapy as an alternative to castration in patients with locally advanced prostate cancer [2].

Therapy with non-steroidal antiandrogens (administrated either as monotherapy or in combination with LHRH agonists) inhibits the effects of circulating testosterone at the androgen receptor level in prostate cells [3,4]. The hormonal feedback mechanism increases the secretion of luteinizing hormone from the pituitary gland and consequently the secretion of testosterone from the testes [3]. Non-suppressed testosterone levels are important for sexual function. However, increased levels of circulating testosterone also result in higher estrogen levels because testosterone is peripherally aromatized to estrogen. This process stimulates the growth of breast tissue, causing so-called breast events, such as gynecomastia and breast pain [5]. In the Early Prostate Cancer trial, gynecomastia and breast pain developed in 69% and 74% of 4,022 randomized patients, respectively, treated with non-steroidal antiandrogens (bicalutamide 150 mg daily) within the first six to nine months, and these adverse events were the major reasons for discontinuation of therapy [6,7]. After diagnosis of gynecomastia or breast pain the aim is to treat the symptoms and to prevent further breast enlargement using radiotherapy to the breast tissue, medical or surgical treatments. However, it is unclear how many patients in this situation decide to undergo one of these therapy options. Tamoxifen is an antiestrogen that has emerged as a potential therapy for the management of gynecomastia and breast pain [8-11]. Recent studies presented data that tamoxifen might decrease the incidence of breast events [12] and could lead to a complete resolution of gynecomastia [13]. It is considered an alternative to surgical treatment or radiotherapy to the breast tissue, and its use has been discussed widely in the past [14-16]. However, no systematic reviews based on a comprehensive literature search using predefined methodology have yet evaluated the benefits and potential harms of tamoxifen in comparison to other treatment options for either the prophylaxis or treatment of breast events induced by non-steroidal antiandrogens in prostate cancer patients.

## Methods

The review protocol was prospectively registered in the International Prospective Register of Systematic Reviews (http://www.crd.york.ac.uk/PROSPERO; CRD42011001320). We considered and searched (see below) parallel group, randomized controlled trials (RCTs) comparing tamoxifen with any other therapy for the management of breast events induced by non-steroidal antiandrogens in patients with prostate cancer. We imposed no limitations on the ethnicity of the patients but excluded patients with neoplasms other than prostate cancer or with breast events induced by other diseases (such as, alcoholism). We evaluated the effects of tamoxifen both in preventive and therapeutic settings and assessed the following outcomes: gynecomastia, breast pain, the incidence of adverse events and discontinuation due to adverse events.

We initially searched the Cochrane Library (CENTRAL, Issue 6), Ovid MEDLINE (1946 to June 2011) and EMBASE (1947 to June 2011) electronically, and we updated our search on April 7, 2012. The search strategy was adapted for each electronic database [See Additional file 1, Table S1], and no language restrictions were applied. We manually screened the reference lists of all of the identified papers, and in June 2011 we searched the abstracts of papers presented at the conferences of (a) the American Society of Clinical Oncology http://jco.ascopubs.org, (b) the European Association of Urology http://www.uroweb.org and (c) the American Urological Association http://www.abstracts2view.com/aua/ and updated the search on April 7, 2012. In addition, we searched the following trial registers for ongoing or completed studies: Current Controlled Trials (http://www.controlled-trials.com, search updated on April 7, 2012), ClinicalTrials.gov (http://www.clinicaltrials.gov, search updated on April 7, 2012), and the search portal of the WHO (http://www.who.int/ictrp/en/, search updated on April 7, 2012).

One author (FK) screened all of the identified references, and only citations that were clearly irrelevant were excluded at this stage. Two review authors (FK, BK) then examined the full-text reports, identified potentially relevant studies, assessed the eligibility of studies for inclusion, extracted relevant data and assessed the risk of bias of each study. We resolved any disagreement on the eligibility of studies through double-checking the respective reports, discussion between the two authors and, if necessary, the help of a third party (JM).

We used RevMan version 5.1, which was provided by the Cochrane Collaboration http://www.cochrane.org, for the statistical analysis of the data and calculated risk ratios (RRs) with their 95% CIs for the chosen outcomes. We assessed statistical heterogeneity (Chi^2^, I^2^) and used a fixed-effect-model for I^2 ^< 50% and in addition, a random-effects-model as sensitivity analysis if I^2 ^> 50%. All of the statistical tests were two-sided, and *P *< 0.05 was considered statistically significant.

## Results

**Figure 1:**
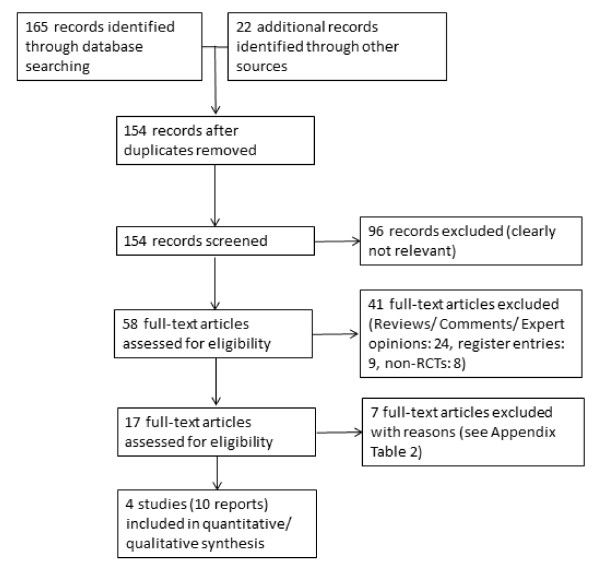
**Search flow chart (June 2011)**.

A total of four studies published in 10 reports fulfilled our inclusion criteria for this review. These studies were published by Boccardo *et al. *[8,17-19] (Boccardo 2005), Fradet et al. [12,20] (Fradet 2007), Perdona *et al. *[9,21] (Perdona 2005), and Saltzstein *et al. *[13,22] (Saltzstein 2005). For the details of the search results, see Figure 1, Table 1 and Table 2. We identified no ongoing studies and no studies that compared tamoxifen with surgical therapies. We identified no additional study with our updated search.

**Table 1:** Study characteristics.

	Boccardo 2005	Fradet 2007	Perdona 2005	Saltzstein 2005
Design	RCT, three arms	RCT, six arms^b^	RCT, three arms	RCT, three arms
Intervention	tamoxifen 20 mg/d (37 patients)	tamoxifen 20 mg/d (35 patients)	tamoxifen 10 mg/d (50 patients)	tamoxifen 20 mg/d (35 patients)
Control	- anastrozole 1 mg/d (36 patients);	- placebo (60 patients)^b^	- radiotherapy (single fraction of 12 Gy, (50 patients);	- anastrozole 1 mg/d (36 patients);
	- placebo (40 patients)		- no additional therapy (51 patients)	- placebo (36 patients)
Assessment of gynecomastia	by breast ultrasound or caliper. ("Severity was scored on the basis of the largest diameter as follows: grade 1, ≤ 2 cm; grade 2, more than 2 to ≤ 4 cm; grade 3, more than 4 to ≤ 6 cm; and grade 4, more than 6 cm.")	by patient questioning and calipers ("recorded in centimeters to the nearest 0.5 cm")	by calipers ("Severity was scored on the basis of the largest diameter: grade 1 (≤ 2 cm); grade 2 (from 2 to ≤ 4 cm); grade 3 (from 4 cm to ≤ 6 cm); and grade 4 (> 6 cm))"	by physical examination and direct patient questioning ("Criteria for a response to randomized therapy was the complete absence of gynecomastia and/or breast pain.")
Assessment of breast pain	by direct patient questioning at each visit ("scored according to severity as none, mild to moderate, or severe")^a^	by direct patient questioning ("rated as mild (awareness of signs or symptoms but easily tolerated), moderate (discomfort sufficient to cause interference with normal activities), or severe (incapacitating resulting in an inability to perform normal activities")	by direct patient questioning at each visit ("scored as none, mild, moderate, or severe")	by direct patient questioning ("Criteria for a response to randomized therapy was the complete absence of gynecomastia and/or breast pain.")
Random sequence generation	randomization lists for each center	computer random number generator	permuted randomization algorithm	sequential order, numbers were not reused, schedule prepared at each center
Allocation concealment	unclear	central allocation	central allocation	central allocation
Blinding of participants/personnel	double-blind, placebo-controlled	double-blind, placebo-controlled	no	double-blind, placebo-controlled
Blinding of outcome assessment	double-blind, placebo-controlled	double-blind, placebo-controlled	no or not mentioned	double-blind, placebo-controlled
Incomplete outcome data	low risk of bias^c^	low risk of bias^c^	low risk of bias^c^	low risk of bias^c^
Selective reporting	low risk of bias^d^	low risk of bias^d^	low risk of bias^d^	low risk of bias^d^
Other remarks	recruitment was stopped early because of planned interim analysis, research funding by AstraZeneca (no role in study design, analysis or interpretation of data)	co-author is an employee of AstraZeneca, writing support funded by AstraZeneca	authors declared no conflict of interest	co-authors are employees of AstraZeneca, no conflict of interest mentioned in manuscript

^a^We included the following pain degrees: mild to moderate, severe; ^b^This study compared multiple dosages of tamoxifen (1, 2.5, 5, 10, or 20 mg daily) with placebo. In this review, we included only the groups treated with tamoxifen 20 mg compared with placebo; ^c^We found no evidence for missing outcome data. Additionally, outcome data were presented by intention-to-treat; ^d^The study protocol is not available, but we assume that the published reports include all evaluated outcomes. RCT, randomized controlled trial.

**Table 2:** Excluded studies with reasons for exclusion.

Authors	Reason for exclusion
Bedognetti *et al. *[23,24]	Study did not evaluate our predefined comparison^a^
Boccardo *et al. *[37]	No relevant topic^b^
Eaton *et al. *[25]	Only abstract available and data not sufficiently detailed to include in review^c^
Parker *et al. *[11]	No prostate cancer patients
Serretta *et al. *[38,39]	Study did not evaluate our predefined comparison^d^

^a^Tamoxifen 20 mg daily versus tamoxifen 20 mg once weekly; ^b^Evaluation of insulin-like growth factor 1 and binding proteins in prostate cancer; ^c^Tamoxifen 20 mg once weekly versus no additional therapy; ^d^Tamoxifen 20 mg/daily given at the early onset of gynecomastia (within one month) for a period of one year versus Tamoxifen 10 mg/daily given for one year starting at the bicalutamide prescription.

All of the included studies were multicenter trials. The number of centers ranged from 5 (Perdona 2005) to 27 (Fradet 2007); the centers were located in either Europe (Boccardo 2005, Fradet 2007, Perdona 2005) or North America (Saltzstein 2005, Fradet 2007). Information on the study populations was provided for all of the trials. The trials included patients with localized or locally advanced prostate cancer treated by local therapy (Fradet 2007, Perdona 2005, Saltzstein 2005), patients who were not suitable for local therapy or patients who had refused such treatments (Bedognetti 2010, Boccardo 2005). One study also included patients presenting with recurrent disease after primary therapy (Boccardo 2005). However, none of the studies included patients with metastatic disease. All of the patients were treated with bicalutamide 150 mg daily as the androgen-suppression therapy. Reporting on detailed characteristics of the study methodology was limited for all of the studies. In one study (Boccardo 2005), patient recruitment was stopped after interim analysis because of a higher incidence of breast events in the control groups. For details on study characteristics and risk of bias, see Table 1. We minimized the impact of possible publication bias in conducting electronic and manual searches of multiple databases without language restriction but were not able to perform a test for funnel plot asymmetry because we did not identify at least 10 studies.

### Prevention of breast events

All four studies (Fradet 2007, Perdona 2005, Boccardo 2005, and Saltzstein 2005) provided data on the prevention of gynecomastia and on breast pain for tamoxifen (10 mg or 20 mg daily) compared to no additional therapy or to placebo. Tamoxifen significantly reduced the risk of suffering from gynecomastia at 3 (RR 0.06, 95% CI 0.01 to 0.43), 6 (RR 0.10, 95% CI 0.05 to 0.22) and 9 to 12 (RR 0.17, 95% CI 0.09 to 0.31) months compared to untreated controls (Table 3). Tamoxifen also significantly reduced the risk of enduring breast pain at 3 (RR 0.09, 95% CI 0.03 to 0.24), 6 (RR 0.06, 95% CI 0.02 to 0.17) and 9 to 12 (RR 0.13, 95% CI 0.06 to .27) months compared to untreated controls (Table 3). Additionally, a sensitivity analysis using a random-effects model for the prevention of breast pain at three months revealed (I^2 ^74%) significant results favoring tamoxifen compared to untreated controls (RR 0.10, 95% CI 0.01 to 0.90).

**Table 3:** Prevention of breast events.

Outcome	Studies	Participants	Risk Ratio (M-H, Fixed, 95% CI), I^2^
Tamoxifen (10 or 20 mg daily) versus no therapy/placebo			
Prevention of gynecomastia			
• at 3 months	Fradet 2007	94	0.06 (0.01, 0.43), -
• at 6 months	Fradet 2007, Perdona 2005	195	0.10 (0.05, 0.22), 0%
• at 9 to 12 months	Boccardo 2005, Fradet 2007	171	0.17 (0.09, 0.31), 0%
Prevention of breast pain			
• at 3 months	Fradet 2007, Saltzstein 2005	165	0.09 (0.03, 0.24), 74%^a^
• at 6 months	Fradet 2006, Perdona 2005	195	0.06 (0.02, 0.17), 27%
• at 9 to 12 months	Boccardo 2005, Fradet 2007	171	0.13 (0.06, 0.27), 0%

Tamoxifen (20 mg daily) versus anastrozole (1 mg daily)			
Prevention of gynecomastia			
• median 12 months	Boccardo 2005	73	0.22 (0.08, 0.58), -
Prevention of breast pain			
• median 12 months	Boccardo 2005, Saltzstein 2005	143	0.25 (0.10, 0.64), 0%

Tamoxifen (10 mg daily) versus radiotherapy			
Prevention of gynecomastia			
• at 6 months	Perdona 2005	100	0.24 (0.09, 0.65), -
Prevention of breast pain			
• at 6 months	Perdona 2005	100	0.20 (0.06, 0.65), -

^a^Risk ratio with random effects model (M-H, 95% CI) 0.10 (0.01 to 0.90).

Two studies (Boccardo 2005 and Saltzstein 2005) reported data on the prevention of gynecomastia or breast pain with tamoxifen (20 mg daily) versus the aromatase inhibitor anastrozole (1 mg daily, see Table 3). One study (Boccardo 2005) showed a significant benefit of tamoxifen in the prevention of gynecomastia after a median of 12 months (RR 0.22, 95% CI 0.08 to 0.58), and two studies (Boccardo 2005 and Saltzstein 2005) provided data on the prevention of breast pain, demonstrating a statistically significant difference favoring tamoxifen after a median of 12 months (RR 0.25, 95% CI 0.1 to 0.64).

One study (Perdona 2005) reported data on the prevention of gynecomastia or breast pain with tamoxifen (10 mg daily) versus radiotherapy (single fraction of 12 Gy), showing significant benefit of tamoxifen for the prevention of gynecomastia (RR 0.24, 95% CI 0.09 to 0.65) and for the prevention of breast pain at six months, respectively (RR 0.2, 95% CI 0.06 to 0.65, Table 3).

### Treatment of breast events

One study (Saltzstein 2005) showed a significant difference favoring tamoxifen for the treatment of breast events (including gynecomastia/breast pain) at three months (RR 0.38, 95% CI 0.25 to 0.58, Table 4), and Perdona *et al. *(Perdona 2005) reported that tamoxifen significantly improved the symptoms of gynecomastia, breast pain or both compared with radiotherapy at nine months (RR 0.21, 95% CI 0.05 to 0.83, Table 4). Perdona *et al. *presented data for patients with grade 3-4 gynecomastia (> 4 cm) who experienced a change of disease to grade 1-2 gynecomastia (≤ 4 cm) [9] and Saltzstein *et al. *reported data for the time to resolution of breast events [13].

**Table 4:** Treatment of breast events.

Outcome	Studies	Participants	Risk Ratio (M-H, Fixed, 95% CI), I^2^
Tamoxifen (20 mg daily) versus anastrozole (1 mg daily)			
Treatment of breast events			
• at 3 months	Saltzstein 2005	90	0.38 (0.25, 0.58), -

Tamoxifen (10 mg daily) versus radiotherapy			
Treatment of gynecomastia/breast pain/or both			
• after 9 months	Perdona 2005	35	0.21 (0.05, 0.83), -

### Tolerability of tamoxifen

Two studies (Fradet 2007 and Saltzstein 2005) reported data on discontinuation due to adverse events. However, only Saltzstein *et al. *specified the adverse events that led to discontinuation (gynecomastia: two; breast pain: five; moderate rise of liver enzymes: one). [13] There were no significant differences between tamoxifen 20 mg daily and placebo (RR 0.92, 95% CI 0.38 to 2.23, Additional file 1, Table S2) or between tamoxifen 20 mg daily and anastrozole 1 mg daily with regard to discontinuations (RR 0.86, 95% CI 0.29 to 2.55, Additional file 1, Table S3). This was, however, probably due to low numbers of discontinuations.

All of the included studies (Boccardo 2005, Saltzstein 2005, Perdona 2005 and Fradet 2007) presented data on adverse events for tamoxifen (10 mg or 20 mg daily) versus no additional therapy or placebo. There were no significant differences for any adverse events [See Additional file 1, Table S2, all *P *> 0.05]. Two studies (Boccardo 2005 and Saltzstein 2005) reported data on adverse events for tamoxifen (20 mg daily) compared to anastrozole (1 mg daily, Additional file 1, Table S3). In total, fewer adverse events occurred with tamoxifen (RR 0.51, 95% CI 0.31 to 0.82), but there were no significant differences for individual adverse events [See Additional file 1, Table S3, all *P *> 0.05]. A single fraction of 12 Gy, compared to tamoxifen 10 mg daily, significantly increased the risk of suffering from nipple erythema (RR 0.11, 95% CI 0.03 to 0.43) and from skin irritation (RR 0.03, 95% CI 0.00 to 0.41) but not the risk for other adverse events [See Additional file 1, Table S4, all *P *> 0.05]. The authors noted that all radiotherapy-associated adverse events resolved and were of short duration (median 4 weeks; Perdona 2005).

## Discussion

We evaluated the efficacy of tamoxifen for the management of breast events induced by non-steroidal antiandrogens in patients with prostate cancer. Our findings suggest that tamoxifen is more effective for both the prevention and treatment of gynecomastia and breast pain compared to other therapies, such as radiotherapy or the selective aromatase inhibitor anastrozole.

We included data from studies evaluating tamoxifen compared to any other therapy for the management of breast events. The criteria for diagnosis of gynecomastia, however, varied among the included studies. The assessment was performed by either examination or patient questioning. This discrepancy could have led to different incidences of breast events in the studies. Additionally, the grading of the severity of breast pain was different in the included studies and ranged from no pain to severe pain. However, breast pain was assessed in all of the studies by direct patient questioning, and even if the severity of breast events is reported to be moderate, this disease is often a reason for patients withdrawing from therapy.

We included tamoxifen with dosages of 10 mg or 20 mg daily in the meta-analysis because Fradet *et al. *found no significant differences in their dose-response study when tamoxifen 20 mg daily was compared to 10 mg daily [12]. However, we included only studies using tamoxifen continuously without interruption. Therapy with tamoxifen is likely to be most effective if it is administered continuously. Bedognetti *et al. *demonstrated that the beneficial effects of tamoxifen 20 mg daily after eight weeks for the prevention of gynecomastia were only significant when administering the drug continuously as opposed to weekly [23,24]. However, there is also evidence that tamoxifen 20 mg once weekly might be superior to no additional therapy [25]. Conversely, Fradet *et al. *noted that in all of the groups (irrespective of dose), a high incidence (> 90%) of breast events occurred after stopping tamoxifen therapy [12,20].

Our results suggest that tamoxifen has a beneficial effect if compared to no treatment for the prevention of breast events. However, not all patients need prophylaxis to prevent the development of breast events induced by non-steroidal antiandrogen therapy [26,27], and not all patients with gynecomastia require treatment [28]. Therefore, a patient-oriented, pragmatic approach appears reasonable. This approach was also proposed by van Poppel and by Di Lorenzo *et al. *[14,15,27]. Before starting non-steroidal antiandrogen treatment (either with non-steroidal monotherapy or in combination with LHRH analogues), patients should be informed about the likelihood of breast events and about possible prophylactic therapy options. As recommended earlier by Di Lorenzo *et al. *[14,15], we also suggest that the physician could wait for the occurrence of breast events in selected patients. Prophylaxis should be started only if the patient is afraid of developing gynecomastia or breast pain.

Our findings regarding the comparison of tamoxifen versus radiotherapy show a beneficial effect favoring tamoxifen for the prevention of breast events, but these results are based on a single study. There is evidence, however, that radiotherapy is also an effective treatment option for breast events. A randomized trial compared a single dose of radiotherapy (10 Gy) with sham radiotherapy for the prevention of breast events and found a significant difference favoring interventional treatment (*P *< 0.001) [29]. Additionally, a non-randomized, comparative study of 253 patients participating in a Scandinavian trial demonstrated a decreased risk for the development of gynecomastia and breast pain with radiotherapy compared with no additional treatment [30]. An expert recommended using radiotherapy as the therapy of choice for the prevention of breast events [27].

Most adverse events were rare in both groups and we found no significant differences for any single adverse event comparing tamoxifen with anastrozole [See Additional file 1, Table S3]. Only the risks for nipple erythema and skin irritation were increased with radiotherapy compared to tamoxifen. Several retrospective and non-comparative studies have suggested that radiotherapy is an option with few and mild acute side effects and with no long-term adverse events [31-33]. The most common side effect with radiotherapy was reversible skin erythema [34]. However, Nieder *et al. *provided data demonstrating that exposing the heart to prophylactic radiotherapy of the mammillary region might contribute to cardiac side effects [35]. A narrative review evaluating safety and tolerability did not conclude either these cardiac side effects or secondary malignancies or pulmonary events, but it noted that no studies have evaluated the long-term effects of radiotherapy in men [34].

We were not able to identify any studies that compared tamoxifen with surgical therapies, such as subcutaneous mastectomy and/or liposuction. Therefore, we cannot draw any conclusions regarding this potential alternative therapy option. Surgical therapies aim to reduce breast size to a normal body contour and to eliminate painful tissue [15,16,36]. Experts in the field of gynecomastia treatment have suggested that surgical liposuction is a valuable therapy option in very early stages and that it is a simple and acceptable technique [15,16]. Other experts have only considered surgical therapies in patients with reduced quality of life due to breast events or to distinct or long-standing gynecomastia [28,36]. This option appears reasonable because gynecomastia presenting for longer than 12 months is unlikely to resolve due to irreversible changes in the breast tissue [5,36]. However, surgical therapies always have potential side effects, such as infections, necrosis, loss of sensation and postoperative body deformity, and should therefore be reserved as a secondary treatment for selected patients.

Our results show that the antiestrogen tamoxifen is a useful therapeutic option for the prevention and treatment of breast events. However, in addition to the discussion of our results, it should be mentioned that treatment with antiestrogens for hormone-dependent tumors, such as prostate cancer, raises some concerns. On the one hand, blocking the effect of estrogens results in effective prevention of breast events induced by non-steroidal antiandrogens [14]. On the other hand, this hormonal manipulation could increase androgen secretion by blocking the negative feedback control of estrogens [14]. Although several trials have investigated the potential effects of tamoxifen co-administration on prostate-specific antigen (PSA) inhibition and on the levels of sex hormones [8,12,13,18,23-25], none of them presented long-term follow-up data. Therefore, the impact of tamoxifen therapy on outcomes, such as long-term adverse events, progression and survival, remains unclear and should be considered when prescribing this treatment.

We did not identify any studies with long-term follow-ups, and evidence for treatment with tamoxifen is limited because there are only a few studies with few events. Despite its potential limitations, this systematic review provides evidence-based guidance to clinicians on this clinically relevant topic. It demonstrates good efficacy of tamoxifen for the prevention and treatment of breast events induced by non-steroidal antiandrogens. This important question, however, requires more definitive answers, and further research with high-quality RCTs and longer-term follow-up is warranted.

## Conclusions

The currently available evidence shows good efficacy of tamoxifen for either the prophylaxis or treatment of breast events induced by non-steroidal antiandrogens in prostate cancer patients. It should be taken into account that evidence is limited because there are only a small number of studies with few events and only short-term follow-up. Therefore, the impact of tamoxifen therapy on long-term adverse events, disease progression and survival remains unclear. Further research with high-quality RCTs and longer-term follow-up is warranted to investigate the benefits and harms of tamoxifen 10 mg and 20 mg compared with radiotherapy.

## Abbreviations

LHRH: luteinizing hormone releasing hormone; RCTs: randomized controlled trials; RR: risk ratio.

## Competing interests

The authors declare that they have no competing interests.

## Authors' contributions

FK and JM designed the review. FK and BK collected the data for the review. FK, BK and JM participated in analysis of the data that were interpreted by FK, BK, JM and BW. FK, BK, JM, BW and GA participated in writing and revising the manuscript. GA and BW provided general advice on the review. All authors read and approved the final manuscript.

## Pre-publication history

The pre-publication history for this paper can be accessed here:

http://www.biomedcentral.com/1741-7015/10/96/prepub

## Supplementary Material

Additional file 1 **Table S1: Search strategy Table S2: Adverse events: Tamoxifen (10 or 20 mg daily) versus no additional therapy or placebo Table S3: Adverse events: Tamoxifen (20 mg daily) versus anastrozole (1 mg daily) Table S4: Adverse events: Tamoxifen (10 mg daily) versus radiotherapy (single fraction of 12 Gy)**. Click here for file
