# Supplementary material for: Tamoxifen for the management of breast events induced by non-steroidal antiandrogens in patients with prostate cancer: a systematic review
Source: BMC Med. 2012 Aug 28;10:96. doi: 10.1186/1741-7015-10-96 (PMC3464149; doi:10.1186/1741-7015-10-96)
Supplement: Additional file 1 — Table S1: Search strategy Table S2: Adverse events: Tamoxifen (10 or 20 mg daily) versus no additional therapy or placebo Table S3: Adverse events: Tamoxifen (20 mg daily) versus anastrozole (1 mg daily) Table S4: Adverse events: Tamoxifen (10 mg daily) versus radiotherapy (single fraction of 12 Gy). [file 1741-7015-10-96-S1.PDF]

Table S1: Search strategy

**Responsible searcher: Kunath**	
**Last searched: 07.04.2012**	
**Medline (Ovid)**	
1	Prostatic Neoplasms/
2	(prostat* adj3 (cancer* or tumo* or neoplas* or carcinom* or malign*)).tw.
3	Gynecomastia/
4	(breast* adj3 (pain* or tenderness* or swelling* or enlarg*)).tw.
5	(gynecomast* or gynaecomast* or mastodyn*).mp.
6	Tamoxifen/
7	(Tamoxifen* or Soltamox* or Tomaxithen* or Nolvadex* or ICI 47699* or ICI 46474* or ICI 46,474*).mp.
8	1 or 2
9	3 or 4 or 5
10	6 or 7
11	8 and 9 and 10
**Embase**	
1	CT=("PROSTATE TUMOR"; "PROSTATE CANCER"; "PROSTATE ADENOCARCINOMA"; "PROSTATE CARCINOMA")
2	(prostat* and (cancer* or tumo* or neoplas* or carcinom* or malign*))/same sent
3	CT="GYNECOMASTIA"
4	(breast* and (pain* or tenderness* or swelling* or enlarge*))/same sent
5	(gynecomast* or gynaecomast* or mastodyn*)/ same sent
6	CT="TAMOXIFEN"
7	(tamoxifen* or soltamox* or tomaxithen* or nolvadex*)/same sent
8	1 OR 2
9	3 OR 4 OR 5
10	6 OR 7
11	8 AND 9 AND 10
**Cochrane Library**	
1	MeSH descriptor Prostatic Neoplasms explode all trees
2	(prostat* NEAR/3 (cancer* or tumo* or neoplas* or carcinom* or malign*))
3	MeSH descriptor Gynecomastia explode all trees
4	(breast* NEAR/3 (pain* OR tenderness* OR swelling* OR enlarge*))
5	MeSH descriptor Tamoxifen explode all trees
6	(tamoxifen* OR soltamox* OR tomaxithen* OR nolvadex*)
7	(gynecomast* or gynaecomast* or mastodyn*)
8	(1 OR 2)
9	(3 OR 4 OR 7)
10	(5 OR 6)
11	(8 AND 9 AND 10)

Table S2: Adverse events: Tamoxifen (10 or 20 mg daily) vs. no additional therapy or placebo

Outcome	Studies	Participants (N=)	Risk Ratio (M-H, Fixed, 95% CI), I²
discontinuation due to adverse events	Fradet 2007, Saltzstein 2005	166	0.92 (0.38-2.23), 28%
total adverse events	Boccardo 2005	77	0.94 (0.52, 1.70), -
serious adverse events	Boccardo 2005, Saltzstein 2005	148	1.07 (0.33, 3.49), 0%
rash	Boccardo 2005, Saltzstein 2005, Perdona 2005	249	0.64 (0.25, 1.61), 0%
fever	Boccardo 2005, Perdona 2005	178	4.21 (0.48, 36.76), 0%
neurologic events	Boccardo 2005, Perdona 2005	178	0.79 (0.18, 3.47), 9%
cardiovascular events	Boccardo 2005, Perdona 2005	178	1.06 (0.35, 3.15), 0%
respiratory events	Boccardo 2005	77	3.24 (0.14, 77.06), -
intercurrent infections	Boccardo 2005	77	2.16 (0.20, 22.86), -
myelotoxicity	Boccardo 2005, Perdona 2005	178	0.35 (0.04, 3.30), 0%
hot flashes	Boccardo 2005, Perdona 2005, Fradet 2007	273	1.70 (0.75, 3.86), 0%
breast pain	Saltzstein 2005	71	0.88 (0.74, 1.04), -
gynecomastia	Saltzstein 2005	71	0.77 (0.52, 1.15), -
pharyngitis	Saltzstein 2005, Fradet 2007	166	1.00 (0.41, 2.44), 0%
vasodilation	Saltzstein 2005	71	2.57 (0.53, 12.39), -
accidental injury	Saltzstein 2005	71	3.09 (0.34, 28.26), -
asthenia	Fradet 2007, Saltzstein 2005, Perdona 2005	267	0.62 (0.27, 1.39), 0%
back pain	Saltzstein 2005	71	7.19 (0.39, 134.39), -
dizziness	Saltzstein 2005, Fradet 2007	166	2.93 (0.91, 9.43), 0%
sinusitis	Saltzstein 2005	71	7.19 (0.39, 134.39), -
constipation	Saltzstein 2005, Perdona 2005, Fradet 2007	267	0.98 (0.42, 2.29), 21%
decreased libido	Saltzstein 2005	71	2.06 (0.20, 21.68), -
diarrhea	Saltzstein 2005, Perdona 2005, Fradet 2007	267	1.62 (0.63, 4.16), 0%
dyspnea	Saltzstein 2005	71	5.14 (0.26, 103.37), -
gastritis	Saltzstein 2005	71	5.14 (0.26, 103.37), -
nausea	Saltzstein 2005	71	0.69 (0.12, 3.86), -
pain	Saltzstein 2005	71	1.03 (0.15, 6.90), -
pelvic pain	Saltzstein 2005	71	5.14 (0.26, 103.37), -
periodontal abscess	Saltzstein 2005	71	2.06 (0.20, 21.68), -
Pruritus	Saltzstein 2005, Perdona 2005	172	3.07 (0.64, 14.81), 0%
somnolence	Saltzstein 2005	71	5.14 (0.26, 103.37), -
urinary frequency	Saltzstein 2005	71	5.14 (0.26, 103.37), -
urinary retention	Saltzstein 2005	71	5.14 (0.26, 103.37), -
urinary tract disorder	Saltzstein 2005	71	2.06 (0.20, 21.68), -
urinary urgency	Saltzstein 2005	71	5.14 (0.26, 103.37), -
vomiting	Saltzstein 2005	71	5.14 (0.26, 103.37), -
abdominal pain	Saltzstein 2005	71	3.08 (0.13, 73.23), -
paresthesia	Saltzstein 2005	71	0.51 (0.05, 5.42), -
urinary tract infection	Saltzstein 2005	71	3.08 (0.13, 73.23), -
dry skin	Saltzstein 2005	71	0.21 (0.01, 4.13), -
hypercholesterolemia	Saltzstein 2005	71	0.15 (0.01, 2.74), -
hypertension	Saltzstein 2005	71	0.21 (0.01, 4.13), -
peripheral edema	Saltzstein 2005	71	0.21 (0.01, 4.13), -
urticaria	Saltzstein 2005	71	0.21 (0.01, 4.13), -
anemia	Perdona 2005	101	3.06 (0.13, 73.35), -
erectile dysfunction	Fradet 2007	95	0.86 (0.08, 9.11), -

Table S3: Adverse events: Tamoxifen (20 mg daily) vs. anastrozole (1 mg daily)

Outcome	Studies	Participants (N=)	Risk Ratio (M-H, Fixed, 95% CI), I²
discontinuation due to adverse events	Saltzstein 2005	71	0.86 (0.29-2.55), -
total adverse events	Boccardo 2005	73	0.51 (0.31, 0.82), -
serious adverse events	Saltzstein 2005, Boccardo 2005	144	0.82 (0.27, 2.52), 0%
rash	Saltzstein 2005, Boccardo 2005	144	1.01 (0.29, 3.56), 47%
fever	Boccardo 2005	73	1.95 (0.18, 20.53), -
neurologic events	Boccardo 2005	73	0.16 (0.02, 1.28), -
cardiovascular events	Boccardo 2005	73	0.49 (0.13, 1.80), -
respiratory events	Boccardo 2005	73	0.97 (0.06, 14.97), -
intercurrent infections	Boccardo 2005	73	0.49 (0.09, 2.49), -
myelotoxicity	Boccardo 2005	73	0.14 (0.01, 2.60), -
hot flashes	Boccardo 2005	73	0.97 (0.06, 14.97), -
breast pain	Saltzstein 2005	71	0.88 (0.74, 1.04), -
gynecomastia	Saltzstein 2005	71	0.80 (0.54, 1.21), -
pharyngitis	Saltzstein 2005	71	2.06 (0.56, 7.59), -
vasodilation	Saltzstein 2005	71	1.71 (0.44, 6.64), -
accidental injury	Saltzstein 2005	71	7.19 (0.39, 134.39), -
asthenia	Saltzstein 2005	71	0.77 (0.19, 3.20), -
back pain	Saltzstein 2005	71	1.03 (0.22, 4.76), -
dizziness	Saltzstein 2005	71	3.09 (0.34, 28.26), -
sinusitis	Saltzstein 2005	71	3.09 (0.34, 28.26), -
constipation	Saltzstein 2005	71	0.69 (0.12, 3.86), -
deceased libido	Saltzstein 2005	71	2.06 (0.20, 21.68), -
diarrhea	Saltzstein 2005	71	2.06 (0.20, 21.68), -
dyspnea	Saltzstein 2005	71	5.14 (0.26, 103.37), -
gastritis	Saltzstein 2005	71	5.14 (0.26, 103.37), -
nausea	Saltzstein 2005	71	2.06 (0.20, 21.68), -
pain	Saltzstein 2005	71	1.03 (0.15, 6.90), -
periodontal abscess	Saltzstein 2005	71	5.14 (0.26, 103.37), -
pruritus	Saltzstein 2005	71	2.06 (0.20, 21.68), -
somnolence	Saltzstein 2005	71	1.03 (0.15, 6.90), -
urinary frequency	Saltzstein 2005	71	5.14 (0.26, 103.37), -
urinary retention	Saltzstein 2005	71	2.06 (0.20, 21.68), -
urinary tract disorder	Saltzstein 2005	71	1.03 (0.15, 6.90), -
urinary urgency	Saltzstein 2005	71	5.14 (0.26, 103.37), -
vomiting	Saltzstein 2005	71	5.14 (0.26, 103.37), -
abdominal pain	Saltzstein 2005	71	0.51 (0.05, 5.42), -
paresthesia	Saltzstein 2005	71	1.03 (0.07, 15.81), -
urinary tract infection	Saltzstein 2005	71	0.34 (0.04, 3.14), -
increased cough	Saltzstein 2005	71	0.15 (0.01, 2.74), -
peripheral edema	Saltzstein 2005	71	0.34 (0.01, 8.14), -

Table S4: Adverse events: Tamoxifen (10 mg daily) vs. radiotherapy (single fraction of 12 Gy)

Outcome	Studies	Participants (N=)	Risk Ratio (M-H, Fixed, 95% CI), I^2
nipple erythema	Perdona 2005	100	0.11 (0.03, 0.43), -
skin irritation	Perdona 2005	100	0.03 (0.00, 0.41), -
pruritus	Perdona 2005	100	2.00 (0.38, 10.43), -
anemia	Perdona 2005	100	3.00 (0.13, 71.92), -
fever	Perdona 2005	100	1.00 (0.06, 15.55), -
myelotoxicity	Perdona 2005	100	0.20 (0.01, 4.06), -
asthenia	Perdona 2005	100	0.40 (0.08, 1.97), -
cardiovascular events	Perdona 2005	100	1.50 (0.26, 8.60), -
neurological events	Perdona 2005	100	2.00 (0.19, 21.36), -
constipation	Perdona 2005	100	1.25 (0.36, 4.38), -
diarrhea	Perdona 2005	100	2.00 (0.38, 10.43), -
hot flashes	Perdona 2005	100	1.50 (0.26, 8.60), -
